# Ovarian Follicular Atresia of Ewes during Spring Puerperium

**DOI:** 10.1155/2012/638928

**Published:** 2012-04-03

**Authors:** Radoslava Vlčková, Drahomíra Sopková, Ján Pošivák, Igor Valocký

**Affiliations:** ^1^Department of Normal Anatomy, Histology and Physiology, Institute of Physiology, University of Veterinary Medicine and Pharmacy in Košice, 041 81 Košice, Slovakia; ^2^Clinic of Horses, University of Veterinary Medicine and Pharmacy in Košice, 041 81 Košice, Slovakia

## Abstract

The distribution of healthy and atretic follicles on the ovarian surface of improved Valachian ewes 17, 24, and 32 days postpartum is reported in this study. The number of healthy follicles was higher on day 24 postpartum and their mean diameter tended to increase to day 32 (*P* < 0.05) with the greatest diameter of 5 mm. 78–81% of atretic follicles ≥3 mm in diameter was observed where apoptosis began in the follicular cells situated at the follicular cavity. The early atretic follicles are characterized by the presence of mitotic pictures. In one ewe 24 days postpartum, small regressive follicular cysts were observed. Contracting atresia is characterized by thickening of the theca interna even to 190 *μ*m. Progesterone and oestradiol-17*β* concentrations were maintained at relatively low levels, but with no significant difference between the days postpartum.

## 1. Introduction

Folliculogenesis progresses in the postpartum period of ewes similarly as in cows [1–3]. In our latitude (48° 40′ 0′′N), the first ovulation after parturition is determined by many factors and may occur 60–70 days at the earliest after lambing, but generally appears in the autumn mating season. Only a few hundred follicles mature throughout the life of an ewe and the others undergo atresia, which occurs in 99.9% of mammals [4–7]. Follicular atresia in themajority of mammals is primarily induced by programmed cell death or apoptosis of granulose and theca cells. Apoptosis is characterized by the fragmentation of internucleosomal DNA, reduction of cell mass, bubbling of the cytoplasmic membrane, and formation of apoptotic bodies [8]. Apoptosis of the granulose cells relates to imbalance between oestradiol and progesterone (E_2_↓, P_4_ ↑) in the follicular fluid [9–11], which stimulates the atresia formation [7, 12]. The concentration of IGF-I is the crucial factor deciding whether the follicle matures or undergoes atresia [9, 10, 13]. There are three basic types of atresia described in cattle—early, definite, and late [14–16]. Marion et al. [14] indicated some thickening of granulose and theca layers in various types of atresia in cattle, but there are no reports of this in ewes. The aim of this study was to observe the distribution of healthy and atretic follicles on the ovaries of ewes in the spring puerperal period, and to establish whether these parameters relate to thickening of the granulose and theca layers of healthy follicles and specific types of atresia, and with hormonal concentrations in the blood (progesterone, oestradiol-17*β*).

## 2. Materials and Methods

### 2.1. Animals

 The experiments were carried out on a farm in the Low Tatras region, Slovak Republic (48° 40′ 0′′N/19° 30′ 0′′E, altitude 600–1000 m) on ewes of Improved Vallachian breed in the spring puerperal period after drying off—day 17 (*N* = 11), day 24 (*N* = 17), and day 32 (*N* = 16). Some ewes were chosen for ovariectomy from each group. Ewes were 4–10 years old, in medium body condition (BCS 2.5–3.5) and weighed 45–50 kg. In the winter, ewes were fed grass silage, meadow hay and maize grit 750 g per head and day. Halite, mineral licks, and water were given *ad libitum*. All procedures were approved by the Ethical Committee of the University of Veterinary Medicine and Pharmacy, Košice, Slovak Republic.

### 2.2. Blood Collection and Hormone Analyses

Blood was collected on days 17, 24, and 32 after parturition routinely from the jugular vein into 5 mL test tubes and allowed to stand for coagulation at room temperature (18 to 22°C), then centrifuged 15 min at 3000 rpm. Blood serum was then deep-frozen at −20°C and later used for assessment of concentrations of progesterone and oestradiol-17*β*. Concentrations of progesterone in blood serum were assessed using the RIA method (RIA PROGESTERONE REF IM1188; IMMUNOTECH, A Beckman Coulter Co. ISO 9001, USA). Samples were assessed in duplicates. Analytical sensitivity (limit of detection) of progesterone was 0.03 ng/mL, and the intraassay and inter-assay coefficients of variation were ≤5.4% and ≤9.1%, respectively. Results are expressed in ng/mL. Concentrations of oestradiol-17*β* in blood serum were assessed using the RIA method (RIA ESTRADIOL REF IM1663; IMMUNOTECH, A Beckman Coulter Co. ISO 9001, USA). Analytical sensitivity was 4 pg/mL and the intra-assay and inter-assay coefficients of variation were ≤15.1% and ≤14.4% respectively. Results are expressed in pg/mL.

### 2.3. Ovary Sampling

 Laparotomy with the ovariectomy was carried out in field conditions on days 17, 24, and 32 after parturition. The animals were deprived of food for 12–18 hours before surgery. General anaesthesia was induced with sodium pentobarbital [17]. The laparotomy procedure was reported previously [18]. The ovaries were carefully pulled into the operation wound and cut for histological processing.

### 2.4. Histological Processing

 The ovaries were cut into smaller sections and fixed in 10% formalin neutralized with Ca_2_CO_3_. Then they were washed in 1% KOH solution dissolved in 80% ethanol for 5 hours, followed by water washing for 1 hour. Fixed and washed ovarian sections were drained in an increasing line of ethanol, supersaturated with methyl salicylate and benzene paraffin embedded in paraffin and sectioned at 5–7 *μ*. Sections of ovary tissue were stained with Mayer haematoxylin and eosin and some with Azan. The staining procedure was carried out in accordance with Vacek [19]. Stained sections were fixed in Canadian balsam.

### 2.5. Image Processing

 Ovarian sections were studied using the PC System for Image Processing LUCIA-G version 4.71 connected to a PAL GKB CCD camera CC-8603 for light microscopy with ZEISS Axiolab equipment (Carl Zeiss Co., Germany). Every 20th section was evaluated. The numbers and sizes of surface antral follicles were studied, and healthy and various types of atretic follicles (early atresia; definite atresia-collapsing, contracting, cystic; late atresia) according to the criteria described by Marion et al. [14] were detected. The thicknesses of granulose and theca layers in healthy, early atretic, contracting and collapsing atretic follicles were calculated from five measurements of a specific layer perpendicular to the basal membrane. Single layers in late atretic follicles appeared mixed and were not distinguishable, so these follicles were excluded from the measurements. Follicles with cystic atresia were excluded as well, due to the very thin theca layer (<30 *μ*) and reduction of the granulose layer to one row connected into a chain.

### 2.6. Statistical Analysis

 The concentrations of progesterone and oestradiol-17*β* in blood serum, sizes and numbers of healthy and atretic follicles and thicknesses of granulose and theca layers were statistically assessed based on the arithmetic mean and its S.E. Variances between the days were compared using one-way ANOVA with Tukey's posttest (GraphPad Prism 3.0 for Windows, GraphPad Software, San Diego California USA). Statistical significance is marked with a superscript star and defined as *P* < 0.05.

## 3. Results

Progesterone and oestradiol-17*β* concentrations were maintained at relatively low levels and there was no significant difference between the days postpartum (Table 1).

Mean total numbers of follicles and follicles <3 mm and ≥3 mm in diameter are shown in Table 2. Total number of follicles and follicles <3 mm in diameter found on the ovarian surface tended to increase to day 32 postpartum, but the difference was not significant (*P* > 0.05), similarly as the number of follicles ≥3 mm, which was higher on day 24 postpartum.

**Figure 1:**
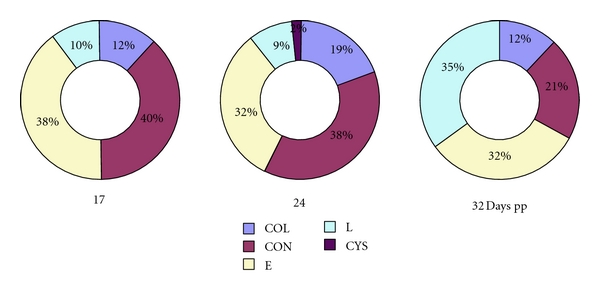
Distribution of follicles in various types of atresia in ewes of Improved Vallachian breed 17, 24, and 32 days postpartum. COL: collapsing atresia, CON: contracting atresia, E: early atresia, L: late atresia, and CYS: cystic atresia; pp: postpartum.

The distribution of healthy and atretic follicles in ewes on days 17, 24, and 32 postpartum is shown in summary in Table 3 and Figure 1. There were no significant differences between numbers of healthy follicles and those in various categories of atresia on the compared days (*P* > 0.05). However, there were 35% of follicles which had undergone late atresia (*P* < 0.05; Figure 1) on day 32 postpartum compared with days 17 and 24. The rate of atresia did not vary significantly between the days (*P* > 0.05; day 17–82%, day 24–84%, and day 32–89%). The number of healthy follicles (Figures 3, and 4) was higher on day 24 postpartum and their mean diameter tended to increase to day 32 (*P* < 0.05) with the greatest diameter of 5 mm. Early atresia (Figure 5) tended to be higher on day 32, and the maximum size of the follicles with this type of atresia was 5.3 mm on day 24. Collapsing atresia (Figure 7) reached the highest number on day 24, similarly as contracting atresia (Figure 6). There was only one cystic follicle (Figure 8) in regression on day 24 with a diameter of 4.31 mm and one with a diameter of 2.8 mm. There were no follicles ≥3 mm in diameter observed with marks of late atresia on any of the studied days.

**Figure 2:**
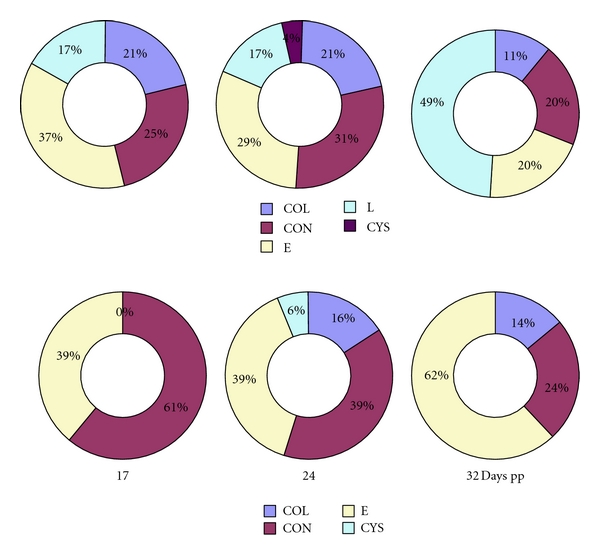
Distribution of follicles <3 mm and ≥3 mm in diameter in various types of atresia in ewes of Improved Vallachian breed 17, 24, and 32 days postpartum. COL: collapsing atresia, CON: contracting atresia, E: early atresia, L: late atresia, and CYS: cystic atresia; pp: postpartum.

**Figure 3:**
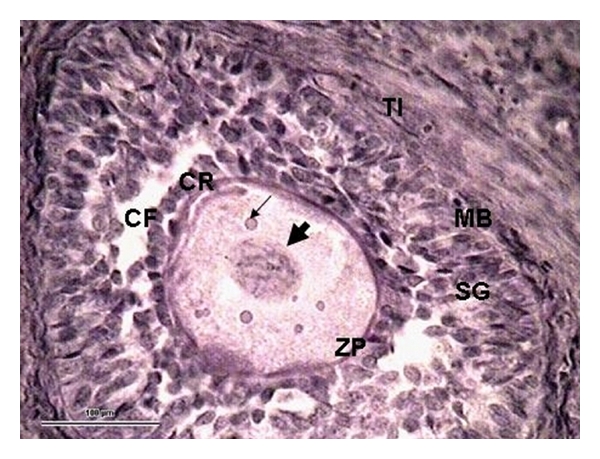
A part of the 0.5 mm tertiary follicle with incipient cavity formation (CF). Follicular cells are placed on a clearly visible *zona pellucida* (ZP) and form *corona radiata* (CR). Fatty drops (arrow) can be seen in the cytoplasm of the oocyte (O) and chromatin in the nucleus (thick arrow). The *membrana basalis* (MB) is clearly visible separating the *stratum granulosum *(SG) from the *theca interna* (TI). (Ewe 32 days postpartum, staining H-E, 100 *μ*m bar, 400x).

**Figure 4:**
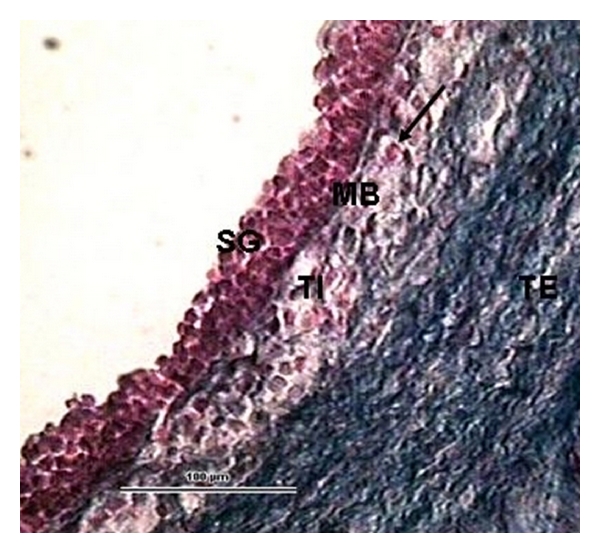
A part of the 4.4 mm preovulatory follicle wall. The *membrana basalis* (MB) is clearly visible separating the *stratum granulosum* (SG) from the *theca interna* (TI), in which glandular cells are clearly differentiated (arrow). The *theca externa* (TE) consists of fibrocytes and vessels. (Ewe 17 days postpartum, staining Azan, 100 *μ*m bar, 400x).

**Figure 5:**
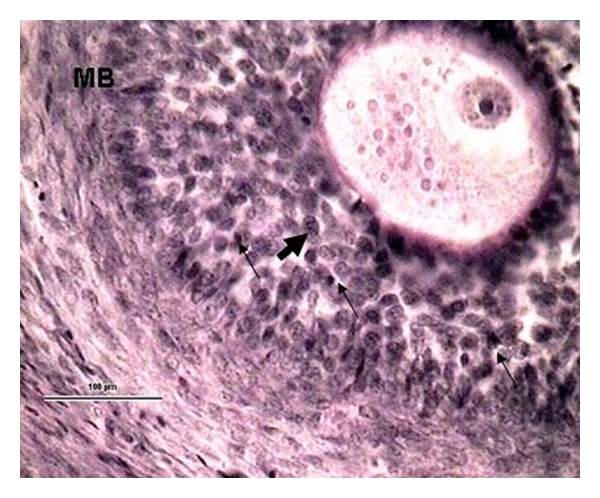
Early atresia of a 0.8 mm follicle with disappearing *membrana basalis* (MB) and with granulation of the oocyte cytoplasm. Numerous cell mitoses (thick arrow) and some atretic bodies (thin arrows) are found in the granulose layer. (Ewe 32 days postpartum, staining H-E, 100 *μ*m bar, 400x).

**Figure 6:**
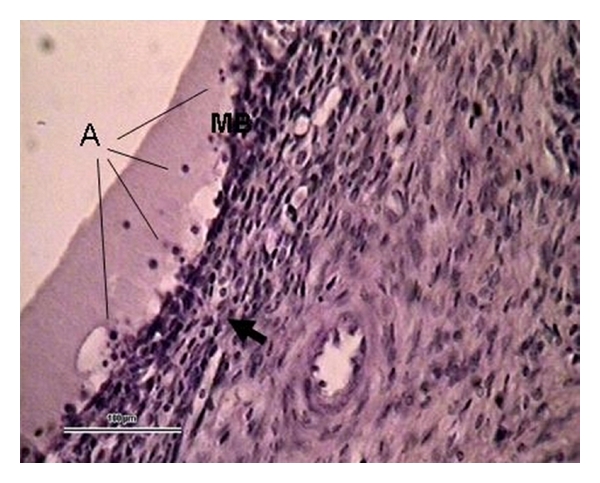
Definitive contracting atresia in a 3.5 mm follicle with granulose cells reduced to one layer, or with degenerated cells forming atretic bodies (A). The basal membrane (MB) is broken, and in some parts it has totally disappeared. Many glandular cells have disappeared from the theca layer, and fibrocytes are shortened and rounded (arrow). (Ewe 17 days postpartum, staining H-E, 100 *μ*m bar, 400x).

**Figure 7:**
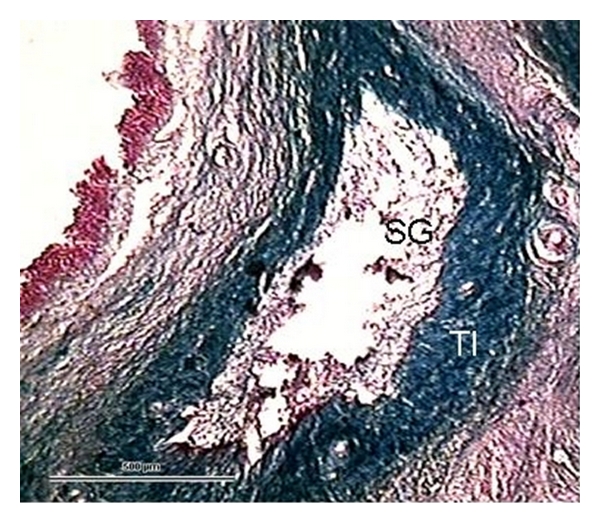
Definitive collapsing atresia (1.4 mm) forming folds, with *theca interna* (TI) totally hyalinised, while the *theca externa* is dedifferentiated and *stratum granulosum* (SG) is fibrotic. (Ewe 17 days postpartum, staining Azan, 500 *μ*m bar, 100x).

**Figure 8:**
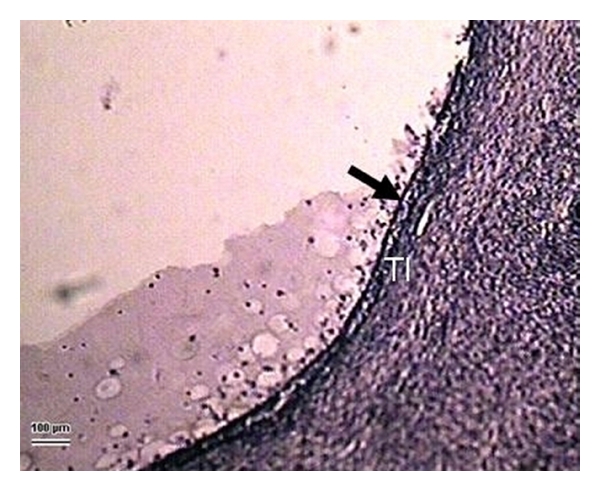
Initial regressive changes in a 4.3 mm follicular cyst. The continuous line of follicular cells (arrow) is broken in some sections, and separating of the individual cells into the follicular cavity is evident. The basal membrane is broken or has disappeared in this phase. Internal theca cells are fibrotic with pycnotic nuclei. The *theca interna* (TI) is only about 30 *μ*m thin. (Ewe 24 days postpartum, staining H-E, 100 *μ*m bar, 100x).

The distribution of healthy and atretic follicles <3 mm and ≥3 mm in diameter in postpartum ewes on days 17, 24, and 32 is shown in Figure 2.

Thicknesses of the granulose and theca layers of healthy and atretic follicles are shown in Table 4. There were no significant differences between the type of atresia, layer and day postpartum. Thicknesses of such layers in healthy follicles did not differ either.

## 4. Discussion

Atresia is particularly specific for the degeneration of the oocyte, follicular cells and hyperplasia of the *theca interna* cells [4, 20]. Pycnosis of the granulose cell nucleus arises and the oocyte loses its round, sharply demarked shape. The *zona pellucida* collapses. Degeneration of the follicular cells appears as chromatolysis, chromatorhexis, fatty, and hyaline degeneration of the ooplasm [8]. The follicular cavity fills with fibroblasts penetrating from the theca layer. The basal membrane forms a hyaline zone. The remnant of the follicle is surrounded by the theca interna cells, which assume the characteristics of epitheloid cells [4, 20]. In ewes, approximately 50–80% of follicles 3-4 mm in diameter are in the early, definitive, and late stage of atresia [20, 21]. In the present study, 78–81% of atretic follicles ≥3 mm in diameter was observed, where apoptosis began in follicular cells situated in the follicular cavity. This is in agreement with the observations of Irving-Rodgers et al. [22], who described this “cavity atresia” in the majority of follicles of all diameters. The results of the present study agree with the authors [23–25], who described the presence of mitotic pictures in early atretic follicles. In one ewe 24 days postpartum, small regressive follicular cysts were observed with the typical follicular cells forming a “strain of pearls” and theca interna <30 *μ*m as described by Marion et al. [14] on the cow ovary. These authors also stated that contracting atresia is characterized by thickening of the theca interna (150 *μ*m), accompanied by shortening and rounding of all the interna cells and disappearance of the glandular internal cells. In the present study, the theca interna was evenly 190 *μ*m thick. Finally, all types of atretic follicles take on the general terminal characteristics [4, 9, 10, 14] of late atresia and disappear.

## 5. Conclusion

The ovaries of ewes in the first month after parturition are in relative inaction, but follicles may develop, mature, and form corpora lutea as during the oestrous period, however of smaller sizes. The rate of atresia of the follicles in stages of recruitment and selection and the size of healthy follicle increase with the length of the postpartum period.

## Figures and Tables

**Table 1:** Mean (± SEM) serum concentrations of progesterone and oeastradiol-17*β* in postpartum ewes of Improved Vallachian breed.

Animals	*N*	Progesterone (ng/mL)	Oestradiol-17*β* (ng/mL)
Ewes 17 days pp	11	0.46 ± 0.04	63.22 ± 5.13
Ewes 24 days pp	17	0.45 ± 0.06	57.43 ± 3.65
Ewes 32 days pp	16	0.51 ± 0.02	57.20 ± 2.81

**Table 2:** Mean (± SEM) numbers of follicles on the ovarian surface in postpartum ewes of Improved Vallachian breed.

Ovaries	*N*	Total *F*	*F* < 3 mm	*F* ≥ 3 mm
Ewes 17 days pp	6	8.50 ± 6.47	4.67 ± 2.89	3.83 ± 1.96
Ewes 24 days pp	8	12.13 ± 5.03	7.25 ± 3.99	4.88 ± 3.14
Ewes 32 days pp	6	14.00 ± 2.75	9.67 ± 2.78	4.33 ± 1.03

**Table 3:** Mean (±SEM) total numbers and sizes of healthy follicles and follicles in various stages of atresia in postpartum ewes of improved Vallachian breed.

Follicles	17 days pp		24 days pp		32 days pp	
	No	Size (mm)	No	Size (mm)	No	Size (mm)
Healthy *F *	1.50 ± 0.84	2.56 ± 1.67*	2.00 ± 1.41	2.73 ± 1.20*	1.50 ± 0.55	3.11 ± 1.46*
Early atresia	2.67 ± 1.63	2.84 ± 1.49	3.25 ± 2.43	2.81 ± 1.27	4.00 ± 0.89	2.44 ± 1.63
Collapsing atresia	0.83 ± 0.41	1.36 ± 0.78	1.88 ± 0.99	2.74 ± 1.34	1.50 ± 0.55	1.87 ± 1.49
Contracting atresia	2.83 ± 0.41	3.17 ± 0.61	3.88 ± 1.13	2.76 ± 0.98	2.67 ± 0.58	2.66 ± 1.22
Cystic atresia	0.00 ± 0.00	0.00 ± 0.00	0.25 ± 0.16	4.31 ± 0.31	0.00 ± 0.00	0.00 ± 0.00
Late atresia	0.67 ± 0.52*	0.96 ± 0.76	0.88 ± 0.35*	0.86 ± 0.47	4.33 ± 1.03*	0.91 ± 0.24

Values within rows: *P < 0.05; pp: postpartum.

**Table 4:** Thicknesses of *stratum granulosum* and *theca interna* of healthy and atretic follicles on the ovaries of ewes in the puerperal period.

Ewes	Layer of the follicular wall	Healthy follicles	Early atresia	Contracting atresia	Collapsing atresia
17 days pp	SG (*μ*m)	107.82 ± 48.65	80.39 ± 24.21	66.52 ± 26.58	52.21 ± 13.17
	TI (*μ*m)	133.32 ± 48.93	171.51 ± 21.87	186.42 ± 2.93	155.43 ± 43.27
24 days pp	SG (*μ*m)	143.57 ± 14.90	97.10 ± 12.55	82.47 ± 18.29	114.56 ± 11.68
	TI (*μ*m)	213.22 ± 18.22	149.74 ± 27.31	191.77 ± 45.58	134.76 ± 14.37
32 days pp	SG (*μ*m)	110.94 ± 5.25	83.18 ± 14.71	63.06 ± 13.19	93.72 ± 14.33
	TI (*μ*m)	146.30 ± 13.63	157.91 ± 31.21	143.24 ± 16.10	119.83 ± 14.03
